# Extended-pulsed fidaxomicin versus vancomycin for *Clostridium difficile* infection: EXTEND study subgroup analyses

**DOI:** 10.1007/s10096-019-03525-y

**Published:** 2019-03-25

**Authors:** Oliver A. Cornely, Maria J. G. T. Vehreschild, Nicholas Adomakoh, Areti Georgopali, Andreas Karas, Gbenga Kazeem, Benoit Guery

**Affiliations:** 1 Department I of Internal Medicine, University Hospital of Cologne and German Centre for Infection Research, Partner Site Bonn-Cologne, 50937 Cologne, Germany; 2 Cologne Excellence Cluster on Cellular Stress Responses in Aging-Associated Diseases (CECAD), Clinical Trials Centre Cologne (ZKS Köln), University of Cologne, Cologne, Germany; 3 Astellas Pharma, Inc., Chertsey, UK; 4 Astellas Pharma Europe Ltd., Chertsey, UK; 5 Astellas Pharma Ltd.,, Chertsey, UK; 6 BENKAZ Consulting Ltd., Cambridge, UK; 7 University Hospital and University of Lausanne, Lausanne, Switzerland

**Keywords:** *Clostridium difficile* infection, Randomised controlled trial, Antibacterial agents, Cohort analyses, Recurrence

## Abstract

Poor outcomes following *Clostridium difficile* infection (CDI) have been associated with advanced age, presence of cancer and *C*. *difficile* PCR-ribotype 027. The impact of baseline risk factors on clinical outcomes was evaluated using data from the EXTEND study, in which rate of sustained clinical cure (SCC) in the overall population was significantly higher with an extended-pulsed fidaxomicin (EPFX) regimen than with vancomycin. Patients aged ≥ 60 years received EPFX (fidaxomicin 200 mg twice daily, days 1–5; once daily on alternate days, days 7–25) or vancomycin (125 mg four times daily, days 1–10). We analysed outcomes by advanced age, cancer diagnosis, CDI severity, prior CDI occurrence and infection with PCR-ribotype 027. The primary endpoint was SCC 30 days after end of treatment (EOT; clinical response at test-of-cure with no subsequent recurrence). SCC rates 30 days after EOT did not differ significantly between EPFX (124/177, 70.1%) and vancomycin (106/179, 59.2%) regardless of age, cancer diagnosis, CDI severity and prior CDI. In patients with PCR-ribotype 027, SCC rate 30 days after EOT was significantly higher with EPFX (20/25, 80%) than with vancomycin (9/22, 40.9%) (treatment difference, 39.1%; 95% CI, 13.2–64.9; *P* = 0.006). Subgroup analyses from the EXTEND study suggest that EPFX is efficacious as a potential treatment for CDI regardless of age, cancer diagnosis, infection with PCR-ribotype 027, CDI severity or prior CDI. ClinicalTrials.gov identifier: NCT02254967.

**Electronic supplementary material:**

The online version of this article (10.1007/s10096-019-03525-y) contains supplementary material, which is available to authorized users.

## Introduction

Outcomes following *Clostridium difficile* infection (CDI) are especially poor in elderly patients, patients with severe disease and those with cancer: these patient groups experience higher rates of CDI complications and/or recurrence [1–4]. Additionally, specific *C*. *difficile* strains linked to infection outbreaks, including PCR-ribotype 027, are associated with severe CDI sequelae compared with other strain types [5–10].

CDI recurrence occurs in approximately 24–27% of cases treated with vancomycin or metronidazole [11] and may be a consequence of disruption and delayed recovery of the natural gut microbiota following antibiotic treatment [12]. Fidaxomicin is associated with both greater conservation of the gut microbiota following CDI treatment [13] and significantly lower recurrence rates than vancomycin [14, 15]. Furthermore, an extended-pulsed fidaxomicin (EPFX) regimen, which extends administration of the 20 tablets of a regular regimen from 10 to 25 days, may enable fidaxomicin to persist in the gut at inhibitory concentrations, thereby suppressing *C*. *difficile* and facilitating microbiota recovery [16].

In the overall EXTEND study population of patients aged ≥ 60 years, EPFX provided significantly superior rates of sustained clinical cure (SCC) compared with standard vancomycin [17]. The present study evaluated clinical outcomes in the EXTEND study population according to presence of cancer, advanced age, CDI severity, prior CDI episodes and presence of *C*. *difficile* PCR-ribotype 027.

## Materials and methods

### Study design and patients

EXTEND was an open-label, randomised, active-comparator controlled, multicentre, phase 3b/4 study conducted in Europe. The study design and primary results have been reported elsewhere [17]. Enrolled patients were aged ≥ 60 years and hospitalised with clinically confirmed CDI, as defined previously [17].

### Treatment and assessments

Patients in the EXTEND study received EPFX (200 mg oral fidaxomicin twice daily on days 1–5, then once-daily administration on alternate days on days 7–25) or vancomycin (125 mg orally, four times daily on days 1–10) [17]. Clinical response, CDI recurrence and safety were assessed as described previously [17]. SCC was defined as clinical response at test-of-cure (TOC) with no subsequent CDI recurrence. PCR ribotyping of all *C*. *difficile* isolates from stool samples was performed at a central laboratory (Leeds Institute of Biomedical & Clinical Sciences, University of Leeds, Leeds, UK), using capillary gel electrophoresis [18, 19].

### Statistical analysis

Efficacy evaluations and analysis sets have been described previously [17]. In the present study, prespecified subgroup analyses assessed efficacy endpoints stratified by the following baseline characteristics: patient age (60–74 years versus ≥ 75 years); cancer diagnosis (presence versus absence); CDI severity (according to European Society of Clinical Microbiology and Infectious Diseases [ESCMID] criteria [5, 17]) and number of prior CDI episodes within 3 months before study participation. A post hoc subgroup efficacy analysis was performed in relation to presence of *C*. *difficile* PCR-ribotype 027 (versus other ribotypes). For each subgroup assessment, the Cochran-Mantel-Haenszel (CMH) test was performed, adjusted for other baseline stratification factors. Common odds ratios, corresponding 95% CIs and descriptive *P* values (significance at ≤ 0.05) were calculated using the CMH test, or the chi-square test for PCR-ribotype assessments.

## Results and discussion

**Fig. 1:**
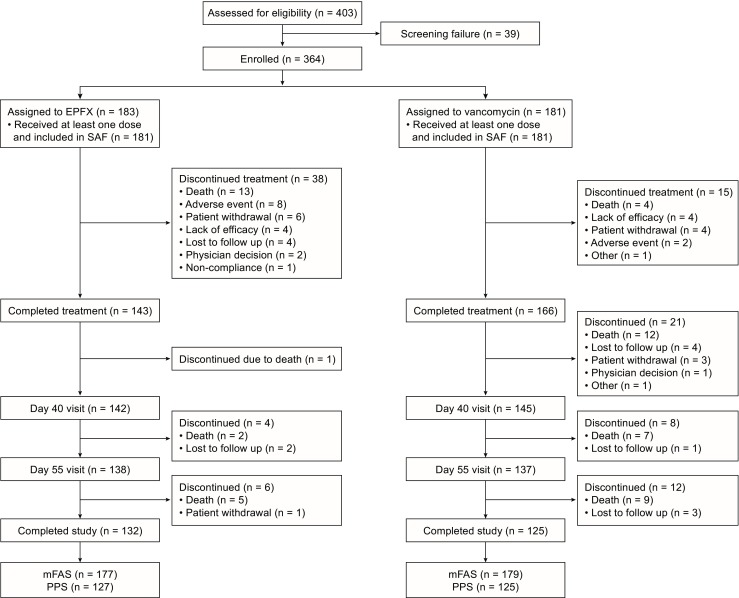
Patient flow through the study

Of the 364 patients randomised (Fig. 1), 356 were included in the modified full analysis set (mFAS, comprising all randomised patients who met the inclusion criteria and received ≥ 1 dose of study medication). The treatment groups had similar baseline characteristics (Table 1) [17].

**Table 1:** Demographics and baseline characteristics of participants, mFAS

Characteristic	EPFX (*n* = 177)	Vancomycin (*n* = 179)	Total (*n* = 356)
Gender, *n* (%)			
Female	107 (60.5)	100 (55.9)	207 (58.1)
Race, *n* (%)^a^			
White	149 (84.2)	153 (85.5)	302 (84.8)
Missing	28 (15.8)	26 (14.5)	54 (15.2)
Median (range) age, years	75.0 (60–94)	75.0 (60–95)	75.0 (60–95)
UBMs per day, *n*^b^			
Mean (SD)	6.8 (4.7)	6.4 (3.4)	6.6 (4.1)
Median	5.0	5.0	5.0
Severe CDI at baseline, *n* (%)^c^	63 (35.6)	67(37.4)	130 (36.5)
Severe CDI by ESCMID score, *n* (%)	78 (44.1)	84 (46.9)	162 (45.5)
No. previous CDI occurrences in the past 3 months, *n* (%)			
0	141 (79.7)	140 (78.2)	281 (78.9)
1	26 (14.7)	29 (16.2)	55 (15.4)
2	10 (5.6)	10 (5.6)	20 (5.6)
Cancer present, *n* (%)^c^	38 (21.5)	37 (20.7)	75 (21.1)
Use of antibiotics for condition other than CDI, *n* (%)			
Yes	128 (72.3)	129 (72.1)	257 (72.2)
Residential setting, *n* (%)	*n* = 175	*n* = 179	*n* = 354
Own residence	102 (58.3)	103 (57.5)	205 (57.9)
Family residence	66 (37.7)	59 (33.0)	125 (35.3)
Nursing home	4 (2.3)	6 (3.4)	10 (2.8)
Long-term care facility	1 (0.6)	4 (2.2)	5 (1.4)
Other	2 (1.1)	7 (3.9)	9 (2.5)
Missing	2	0	2
*C*. *difficile* PCR-ribotype			
027	25 (14.1)	22 (12.3)	47 (13.2)
Other	152 (85.9)	157 (87.7)	309 (86.8)

*EPFX*, extended-pulsed fidaxomicin; *mFAS*, modified full analysis set (all patients with confirmed CDI who were randomised and received at least one dose of study medication); *SD*, standard deviation; *UBMs*, unformed bowel movements

^a^Not all study sites were permitted to report the race of participants; this information was reported as ‘missing’. ^b^In the last 24 h prior to randomisation. ^c^As provided in the Interactive Web Response System at randomisation, and defined as leukocyte count > 15 × 109/L or rise in serum creatinine > 50% above the patient’s normal level or albumin < 30 g/L

### Clinical outcomes

Rates of SCC at 30 days after end of treatment (EOT) (the primary endpoint) did not differ significantly between EPFX and standard vancomycin treatment in most of the subgroups analysed; however, in patients with *C*. *difficile* PCR-ribotype 027, rates were significantly higher with EPFX than vancomycin (Table 2).

**Table 2:** Rates of sustained clinical cure of *Clostridium difficile* infection (CDI) at 30 days after end of treatment with extended-pulsed fidaxomicin (EPFX) and vancomycin in subgroups stratified by baseline characteristics, mFAS

Subgroup	Sustained clinical cure of CDI 30 days after EOT (%; 95% CI)		Treatment difference (95% CI); *P* value
	EPFX (*N* = 177)	Vancomycin (*N* = 179)	
Age ≥ 75 years	66/97 (68.0; 58.8, 77.3)	55/97 (56.7; 46.8, 66.6)	11.3 (− 2.2, 24.9); *P* = 0.224
Age < 75 years	58/80 (72.5; 62.7, 82.3)	51/82 (62.2; 51.7, 72.7)	10.3 (− 4.0, 24.7); *P* = 0.371
Severe CDI	39/63 (61.9; 49.9, 73.9)	34/67 (50.7; 38.8, 62.7)	11.2 (− 5.8, 28.1); *P* = 0.235
Non-severe CDI	85/114 (74.6; 66.6, 82.6)	72/112 (64.3; 55.4, 73.2)	10.3 (− 1.7, 22.2); *P* = 0.068
No previous CDI episode	99/141 (70.2; 62.7, 77.8)	88/140 (62.9; 54.9, 70.9)	7.4 (− 3.6, 18.4); *P* = 0.166
One previous CDI episode	17/26 (65.4; 47.1, 83.7)	14/29 (48.3; 30.1, 66.5)	17.1 (− 8.7, 42.9); *P* = 0.118
Two previous CDI episodes	8/10 (80.0; 55.2, 100.0)	4/10 (40.0; 9.6, 70.4)	40.0 (0.8, 79.2); *P* = 0.141
Cancer present	23/38 (60.5; 45.0, 76.1)	18/37 (48.6; 32.5, 64.8)	11.9 (− 10.5, 34.3); *P* = 0.274
Cancer absent	101/139 (72.7; 65.3, 80.1)	88/142 (62.0; 54.0, 70.0)	10.7 (− 0.2, 21.6); *P* = 0.061
PCR-RT 027	20/25 (80.0; 64.3, 95.7)	9/22 (40.9; 20.4, 61.5)	39.1 (13.2, 64.9); *P* = 0.006
Other RT	104/152 (68.4; 61.0, 75.8)	97/157 (61.8; 54.2, 69.4)	6.6 (− 4.0, 17.2); *P* = 0.221

*CI*, confidence interval; *EOT*, end of treatment; *EPFX*, extended-pulsed fidaxomicin; *mFAS*, modified full analysis set (all patients with confirmed CDI who were randomised and received at least one dose of study medication); *PCR*, polymerase chain reaction; *RT*, ribotype. *P* values were obtained from the Cochran-Mantel-Haenszel test, apart from the *P* value for the difference in outcome for PCR-ribotype 027 versus other ribotypes, which was obtained from the chi-square test. Thirty days after EOT is day 55 for the EPFX arm and day 40 for the vancomycin arm

SCC rates at days 40 and 90 were significantly higher with EPFX than with standard vancomycin in patients aged ≥ 75 years (Online Resource; ESM Fig. 1a). SCC rates at other time points and clinical response rates at day 12 and 2 days after EOT did not differ between treatments for either age category (Online Resource; ESM Fig. 1a and 1b). Our findings are supported by a previous study that used regression modelling to test the effects of age on treatment outcomes in phase 3 trials of standard-regimen fidaxomicin: a significantly higher probability of SCC was shown with fidaxomicin than standard vancomycin (odds ratio, 1.86; 95% CI, 1.40–2.47; *P* < 0.001). However, irrespective of treatment choice, the probability of SCC decreased by 13% and the risk of recurrence increased by 17% for each decade increase in age [20].

At day 40, rates of SCC were significantly higher with EPFX than with vancomycin in both the severe and non-severe CDI subgroups. At days 55 and 90, SCC rates were significantly higher with EPFX than vancomycin only in the non-severe CDI subgroup (Online Resource; ESM Fig. 2a). There were no significant between-treatment differences in efficacy at other time points in patients with severe and non-severe CDI (Online Resource; ESM Fig. 2b).

Patients with a previous history of CDI are at greater risk of developing a further CDI episode [21]. Seventy-five (21%) patients in our analysis had experienced a recent CDI episode prior to study enrolment; the majority (55 [15%]) had a single prior episode. Rates of SCC at days 40, 55 and 90 were significantly higher with EPFX than with vancomycin in patients who had no prior occurrence of CDI. Additionally, rates of SCC at day 40 were significantly higher with EPFX in patients with one or two prior CDI episodes (Online Resource; ESM Fig. 3a). Other efficacy endpoints did not differ between treatments regardless of number of prior CDI episodes (Online Resource; ESM Fig. 3a and 3b). In a previous subgroup analysis, the 28-day SCC rate among patients with one prior CDI episode was 80.3% with standard fidaxomicin and 64.5% with standard vancomycin, higher than the rates observed in our analyses; this difference may be due to the younger age (median 63 years) of the patients in the previous analysis [22]. Our conclusions are also limited by the low number of patients in our study with one or two prior CDI episodes, and the correspondingly high within-sample variability.

Patients without cancer at baseline had significantly higher rates of SCC at days 40, 55 and 90 with EPFX versus vancomycin (Online Resource; ESM Fig. 4a). Other efficacy endpoints in patients with and without cancer did not differ between treatments (Online Resource; ESM Fig. 4a and 4b). These results contrast with those from a previous analysis by Cornely et al. in which the SCC rate was significantly higher with standard-regimen fidaxomicin than vancomycin (73.6% versus 52.1%; *P* = 0.003) in 183 patients with CDI and cancer from two phase 3 studies [2]. This difference in outcome may be attributable to the smaller sample size of our study (75 patients with CDI and baseline cancer) and slightly greater median age (75 years for the overall population in our study versus 69 and 63 years in patients with and without cancer, respectively, in the Cornely et al. study [2]). Our findings aligned with those of the previous study in that patients with cancer had lower rates of initial clinical cure and SCC than patients without cancer, regardless of treatment choice.

**Fig. 2:**
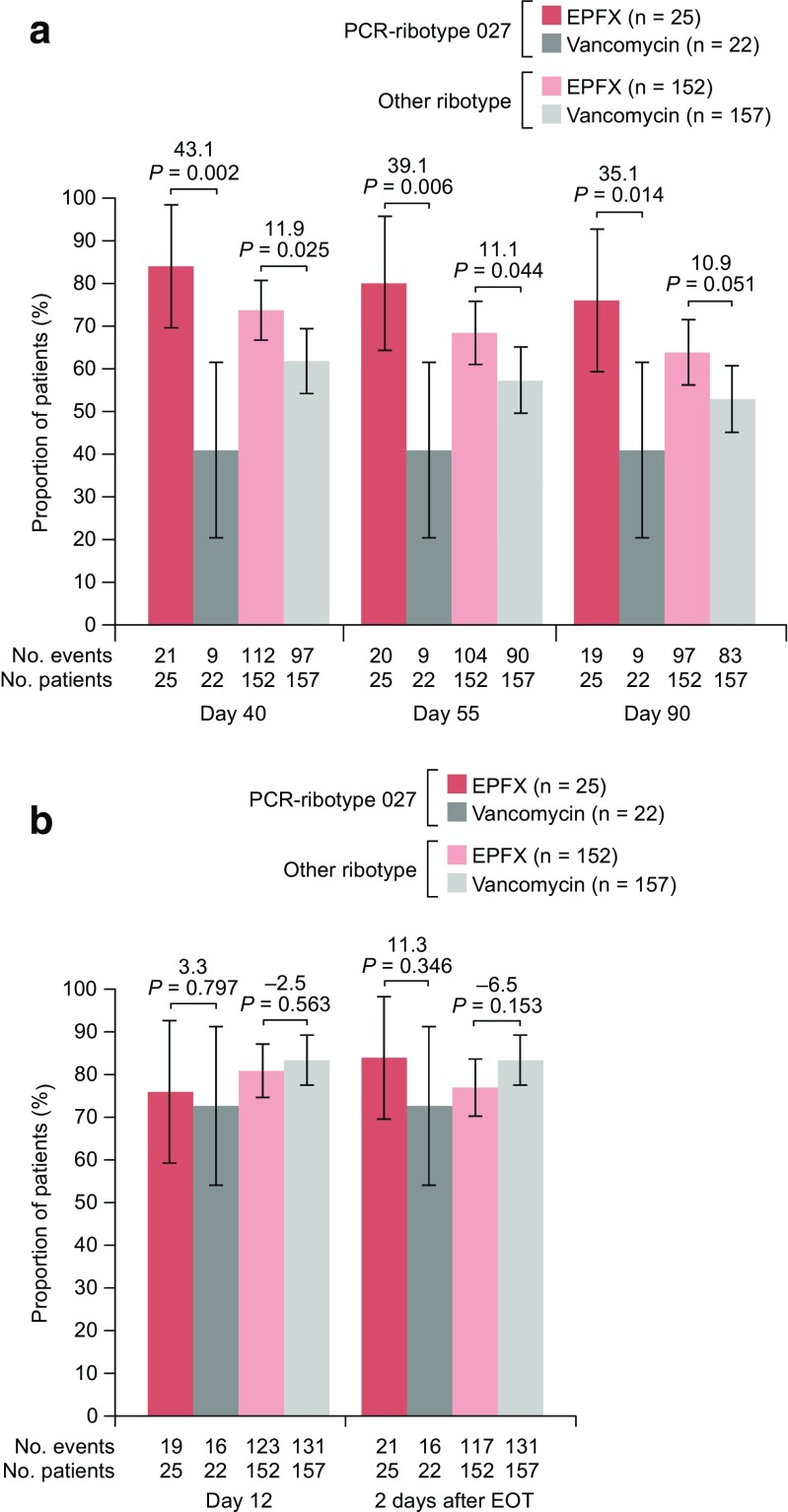
Clinical outcomes of *Clostridium difficile* infection treatment with extended-pulsed fidaxomicin (EPFX) and vancomycin by presence of *C*. *difficile* PCR-ribotype 027 (present or absent), mFAS. **a** Sustained clinical cure (SCC) over time. **b** Clinical response. *EOT*, end of treatment; *mFAS*, modified full analysis set (all patients with confirmed CDI who were randomised and received at least one dose of study medication). *P* values were obtained from the chi-square test. Two days after EOT is day 27 for the EPFX arm and day 12 for the vancomycin arm; 30 days after EOT is day 55 for the EPFX arm and day 40 for the vancomycin arm

*C*. *difficile* PCR-ribotype 027 was the most prevalent ribotype in a recent pan-European survey [23] and has been associated with outbreaks of increased severity [7] and greater risk of CDI recurrence than other strains [20]. In our study, rates of SCC at days 40, 55 and 90 were significantly higher with EPFX than with vancomycin in patients infected with *C*. *difficile* PCR-ribotype 027 (Fig. 2a). Rates of SCC at days 40 and 55 were significantly higher with EPFX than vancomycin in patients with other *C*. *difficile* PCR-ribotypes. No other significant between-treatment efficacy differences were observed in relation to *C*. *difficile* PCR-ribotype (Fig. 2a, b). In previous phase 3 registration studies, rates of recurrence were significantly lower in patients infected with non-PCR-ribotype 027 strains but not in patients infected with PCR-ribotype 027 [14, 15]. Further studies would be required to provide analyses of sufficient power to permit robust conclusions on efficacy differences in relation to PCR-ribotype 027.

Of note, clinical response at day 12 and 2 days after EOT was numerically lower with EPFX than with vancomycin in the majority of subgroups investigated here, and in the overall mFAS population [17]. These results contrast with those of previous phase 3 trials [14, 15], in which standard-regimen fidaxomicin achieved numerically higher rates of clinical cure at 2 days after EOT, compared with standard vancomycin. The lower rate of initial clinical response with EPFX may be due to the administration of fidaxomicin on only alternate days after day 5, thus delaying the reduction in *C*. *difficile* count compared with vancomycin treatment.

### Recurrence of CDI

Current ESCMID guidelines recommend that patients at risk of recurrent CDI are given standard-regimen fidaxomicin or vancomycin [5]. Standard-regimen fidaxomicin is associated with a lower recurrence rate than standard vancomycin [14, 15]; moreover, in the overall EXTEND population, EPFX showed even lower recurrence rates than previously observed with the standard regimen [17]. However, there was no between-treatment difference in the incidence of recurrent CDI according to the subgroups investigated here, although numbers were too small to permit definitive conclusions (Table 3).

**Table 3:** Recurrence of CDI at day 90 stratified by randomisation factors, mFAS

CDI severity	Cancer status	Age (years)	Number of previous occurrences	Recurrence of CDI (%; 95% CI)		Treatment difference (95% CI)
				EPFX (*N* = 138)	Vancomycin (*N* = 147)	
Severe	Presence	≥ 75	2	0/1	0/1	
			1		0/2	
			0	0/5	0/3	
		60–74	2			
			1		0/1	
			0	0/1	1/5 (20.0; 0.5, 71.6)	− 20.0 (− 97.5, 84.8)
	Absence	≥ 75	2		1/1 (100.0; 2.5, 100.0)	
			1	0/4	1/2 (50.0; 1.3, 98.7)	− 50.0 (− 98.7, 45.2)
			0	2/19 (10.5; 1.3, 33.1)	5/24 (20.8; 7.1, 42.2)	− 10.3 (− 39.0, 19.5)
		60–74	2		1/1 (100.0; 2.5, 100.0)	
			1	1/1 (100.0; 2.5, 100.0)		
			0	1/14 (7.1; 0.2, 33.9)	4/10 (40.0; 12.2, 73.8)	− 32.9 (− 66.9, 8.2)
Non-severe	Presence	≥ 75	2	0/1		
			1			
			0	0/6	5/8 (62.5; 24.5, 91.5)	− 62.5 (− 91.5, − 10.1)
		60–74	2			
			1	0/1	0/2	
			0	0/9	1/6 (16.7; 0.4, 64.1)	− 16.7 (− 64.1, 36.2)
	Absence	≥ 75	2	0/5	1/3 (33.3; 0.8, 90.6)	− 33.3 (− 90.5, 37.0)
			1	1/6 (16.7; 0.4, 64.1)	2/6 (33.3; 4.3, 77.7)	− 16.7 (− 69.7, 44.7)
			0	1/27 (3.7; 0.1, 19.0)	3/30 (10.0; 2.1, 26.5)	− 6.3 (− 32.0, 19.5)
		60–74	2	1/3 (33.3; 0.8, 90.6)	0/1	33.3 (− 80.9, 97.5)
			1	1/8 (12.5; 0.3, 52.7)	4/10 (40.0; 12.2, 73.8)	− 27.5 (− 66.8, 19.3)
			0	2/27 (7.4; 0.9, 24.3)	4/31 (12.9; 3.6, 29.8)	− 5.5 (− 30.5, 20.2)

Includes only patients who had clinical response at 2 days after EOT

### Safety

The safety profiles of EPFX and standard vancomycin were broadly similar within the subgroups analysed. A greater number of treatment-emergent adverse events, serious adverse events and deaths were reported in patients aged ≥ 75 years in the vancomycin arm than in the EPFX arm (Table 4).

**Table 4:** Treatment-emergent adverse events (based on MedDRA v14.1) during the study, by subgroup, SAF

TEAE frequency by subgroup	EPFX (*n* = 181)	Vancomycin (*n* = 181)
	Patients, *n* (%)	
Any TEAE		
All patients	121 (66.9)	128 (70.7)
Age category		
60–74 years	56 (30.9)	53 (29.3)
≥ 75 years	65 (35.9)	75 (41.4)
CDI severity		
Severe	50 (27.6)	49 (27.1)
Non-severe	71 (39.2)	79 (43.6)
Prior CDI occurrence		
None	94 (51.9)	101 (55.8)
One	19 (10.5)	18 (9.9)
Two	8 (4.4)	9 (5.0)
Cancer diagnosis		
Present	23 (12.7)	32 (17.7)
Absent	98 (54.1)	96 (53.0)
Any serious TEAE^a^		
All patients	68 (37.6)	78 (43.1)
Age category		
60–74 years	33 (18.2)	30 (16.6)
≥ 75 years	35 (19.3)	47 (26.0)
CDI severity		
Severe	33 (18.2)	33 (18.2)
Non-severe	35 (19.3)	44 (24.3)
Prior CDI occurrence		
None	58 (32.0)	61 (33.7)
One	7 (3.9)	11 (6.1)
Two	3 (1.7)	5 (2.8)
Cancer diagnosis		
Present	17 (9.4)	21 (11.6)
Absent	51 (28.2)	56 (30.9)
TEAE-related death^b^		
All patients	28 (15.5)	36 (19.9)
Day 1–27	17 (9.4)	9 (5.0)
Day 27–95^b^	12 (7.3)	27 (15.7)
Age category		
60–74 years	12 (6.6)	12 (6.6)
≥ 75 years	16 (8.8)	24 (13.3)
CDI severity		
Severe	14 (7.7)	14 (7.7)
Non-severe	14 (7.7)	22 (12.2)
Prior CDI occurrence		
None	25 (13.8)	32 (17.7)
One	1 (0.6)	3 (1.7)
Two	2 (1.1)	1 (0.6)
Cancer diagnosis		
Present	9 (5.0)	13 (7.2)
Absent	19 (10.5)	23 (12.7)

Results are given for the Safety Analysis Set: all patients who were randomised and received at least one dose of study medication. *CDI*, *Clostridium difficile* infection; *TEAE*, treatment-emergent adverse event. ^a^TEAE resulting in death, hospitalisation, persistent or significant disability, incapacity, congenital abnormality, or other medically important events; or considered to be life-threatening. ^b^Includes one death in the EPFX arm not considered TEAE-related as it occurred after day 90

## Conclusions

A strength of the present analysis was the inclusion of elderly patients (median age, 75 years), who are not typically enrolled in randomised, controlled trials because polypharmacy and multiple underlying conditions may obscure potential treatment benefits. However, we successfully demonstrate positive outcomes in this difficult-to-treat population. The extensive 90-day follow-up period of our study also provides considerable opportunity to assess long-term efficacy and safety.

Limitations of the study include the lack of tapered vancomycin or standard 10-day fidaxomicin regimens as comparators, which would have broadened the information available but restricted the feasibility of performing such a study. In addition, the post hoc analysis results in relation to PCR-ribotype were not corrected for multiple testing. However, the results obtained for this and other subgroups were consistent across the assessed endpoints, and adjustment for multiple testing was not expected to impact on proportions and confidence intervals, only *P* values. When statistically significant, the latter were markedly lower than the ≤ 0.05 threshold in most instances. Although the subgroups analysed here are small, the results suggest greater efficacy with EPFX versus vancomycin in some patient groups. Additionally, EPFX was not associated with lower SCC rates or a higher incidence of adverse events, compared with standard vancomycin, in any patient subgroup investigated here.

In summary, subgroup analyses of the EXTEND study determined that an extended-pulsed fidaxomicin regimen is efficacious and well tolerated as a potential treatment for CDI regardless of age, presence of cancer, infection with *C*. *difficile* PCR-ribotype 027, CDI severity or prior CDI episodes.

## Electronic supplementary material

ESM 1 (DOCX 628 kb)

ESM 2 (ZIP 10.8 mb)

## Data Availability

Access to anonymised individual participant level data collected during the trial, in addition to supporting clinical documentation, is planned for trials conducted with approved product indications and formulations, as well as compounds terminated during the development. Conditions and exceptions are described under the Sponsor Specific Details for Astellas on www.clinicalstudydatarequest.com. Study-related supporting documentation is redacted and provided if available, such as the protocol and amendments, statistical analysis plan and clinical study report. Access to participant level data is offered to researchers after publication of the primary manuscript (if applicable) and is available as long as Astellas has legal authority to provide the data. Researchers must submit a proposal to conduct a scientifically relevant analysis of the study data. The research proposal is reviewed by an Independent Research Panel. If the proposal is approved, access to the study data is provided in a secure data sharing environment after receipt of a signed Data Sharing Agreement.
