# Supplementary material for: Extended-pulsed fidaxomicin versus vancomycin for Clostridium difficile infection: EXTEND study subgroup analyses
Source: Eur J Clin Microbiol Infect Dis. 2019 Mar 25;38(6):1187–94. doi: 10.1007/s10096-019-03525-y (PMC6520315; doi:10.1007/s10096-019-03525-y)

# Electronic Supplementary Material: Online Resource 1.

# Clinical outcomes of *Clostridioides* (*Clostridium) difficile* infection treatment with extended-pulsed fidaxomicin (EPFX) and vancomycin by baseline subgroup.

Abbreviations: EOT, end of treatment; EPFX, extended-pulsed fidaxomicin; mFAS, modified Full Analysis Set (all patients with confirmed CDI who were randomized and received at least one dose of study medication). *P* values were obtained from the Cochran-Mantel-Haenszel test. Two days after EOT is Day 27 for the EPFX arm and Day 12 for the vancomycin arm; 30 days after EOT is Day 55 for the EPFX arm and Day 40 for the vancomycin arm

## ESM Fig. 1 Clinical outcomes of *Clostridium difficile* infection treatment with extended-pulsed fidaxomicin (EPFX) and vancomycin by age category (60–74 years or ≥75 years), mFAS. a) Sustained clinical cure (SCC) over time. b) Clinical response.

**a)**


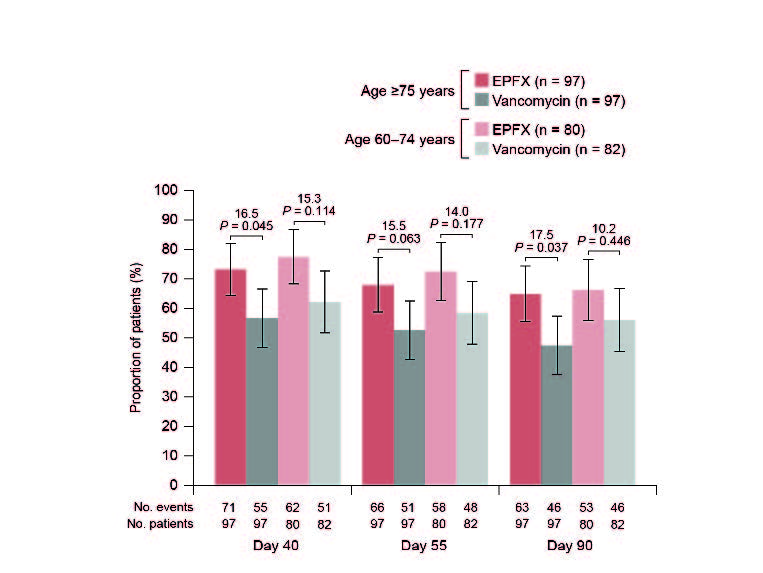


**b)**


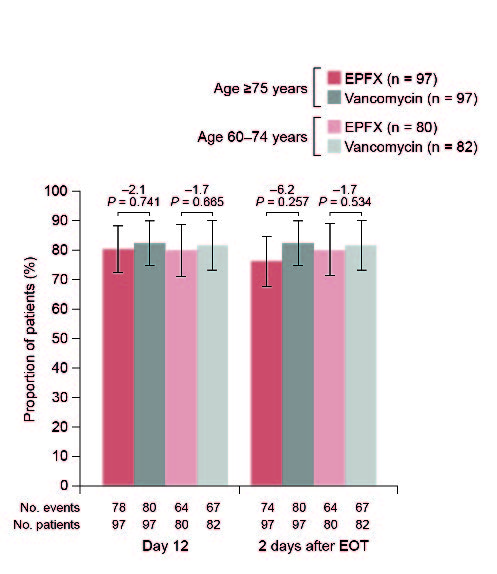


## ESM Fig. 2 Clinical outcomes of *Clostridium difficile* infection treatment with extended-pulsed fidaxomicin (EPFX) and vancomycin by baseline severity of CDI (severe or non-severe), mFAS. a) Sustained clinical cure (SCC) over time. b) Clinical response.

**a)**


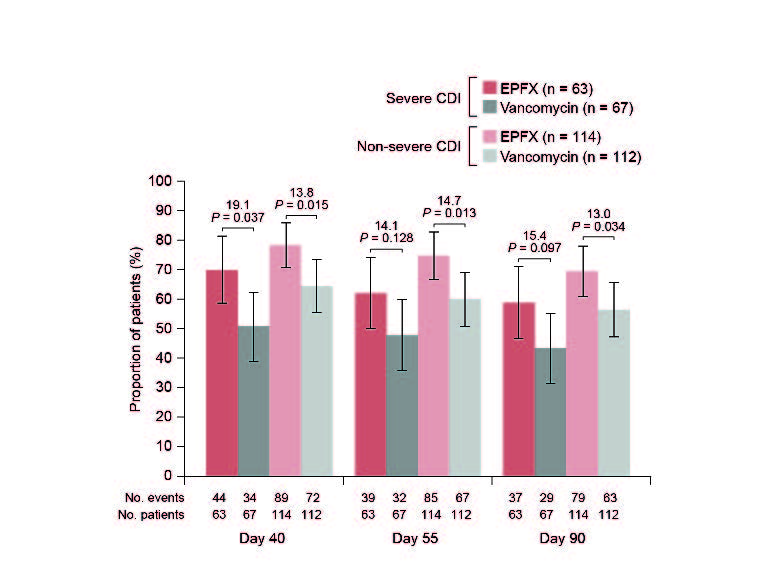


**b)**


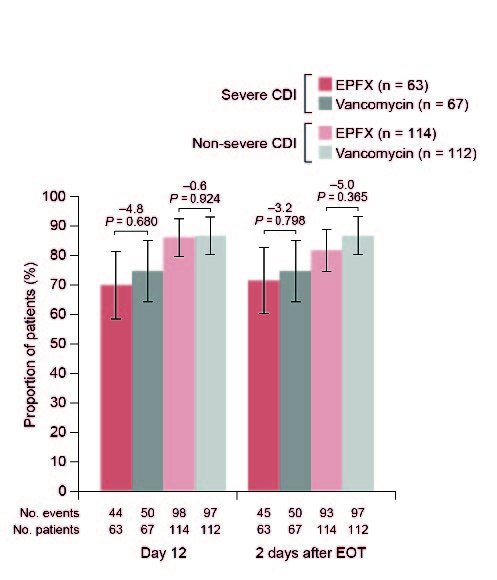


## ESM Fig. 3 Clinical outcomes of *Clostridium difficile* infection treatment with extended-pulsed fidaxomicin (EPFX) and vancomycin by the number of CDI episodes (0, 1, or 2) in the 3 months before the study, mFAS. a) Sustained clinical cure (SCC) over time. b) Clinical response.

**a)**


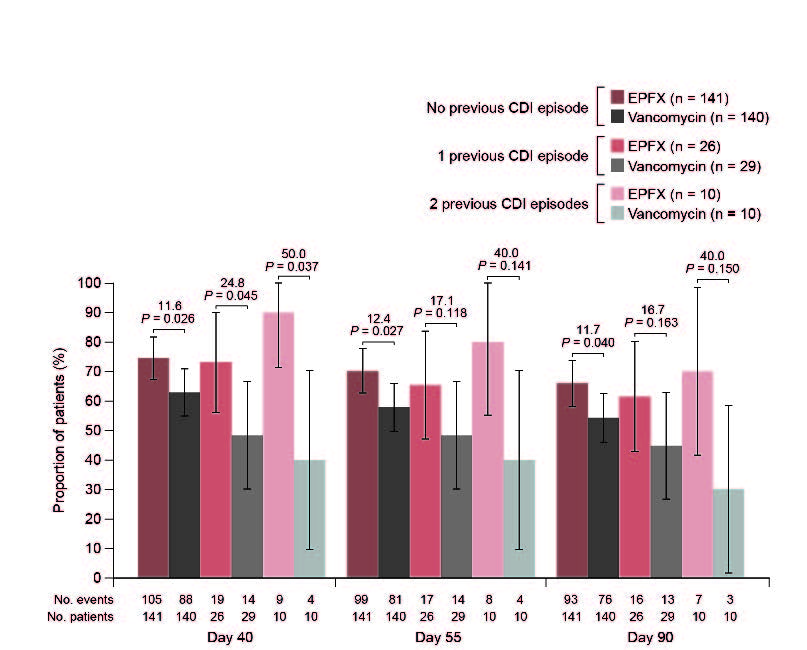


**b)**


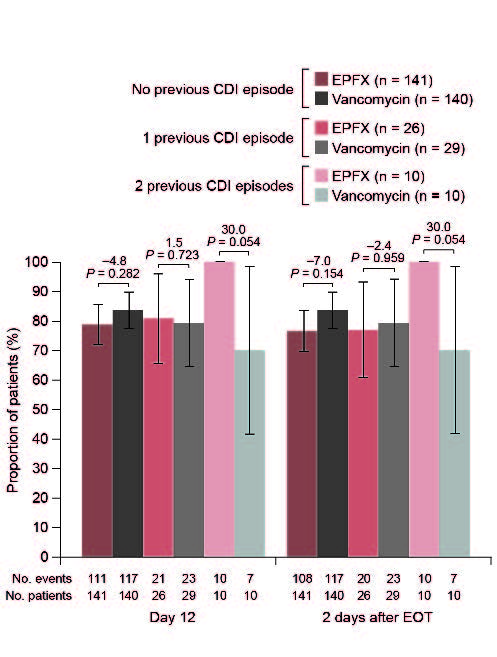


## ESM Fig. 4 Clinical outcomes of *Clostridium difficile* infection treatment with extended-pulsed fidaxomicin (EPFX) and vancomycin by baseline cancer diagnosis (presence or absence), mFAS. a) Sustained clinical cure (SCC) over time. b) Clinical response.

**a)**

**
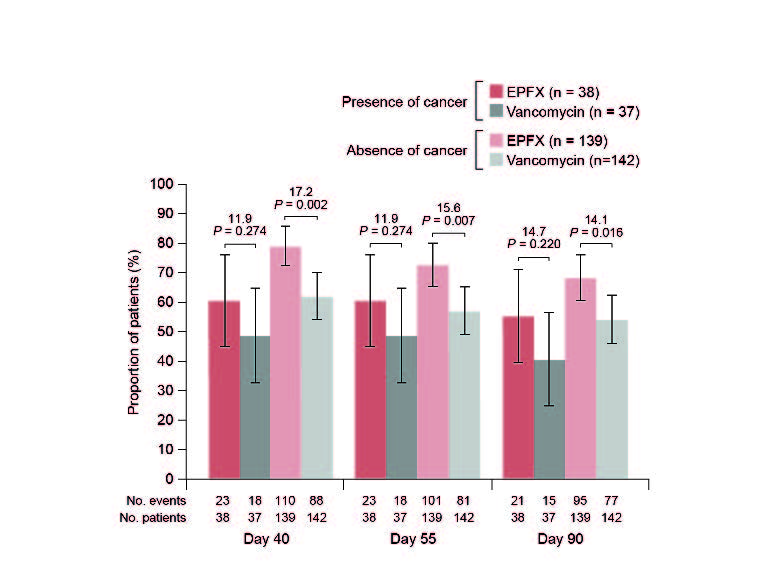
**

**b)**


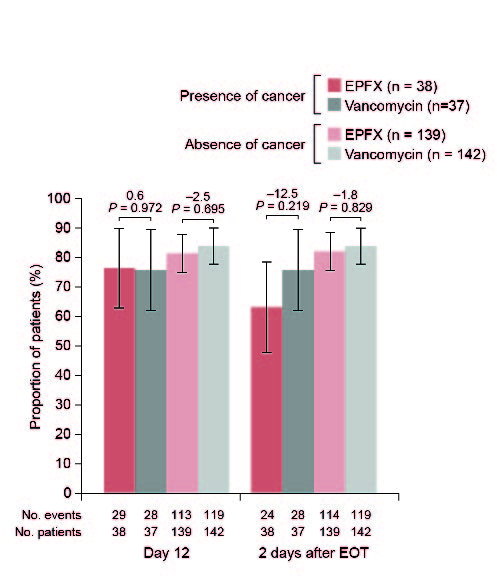

Supplement: Supplementary file 1 — (DOCX 628 kb) [file 10096_2019_3525_MOESM1_ESM.docx]
